# Pioneering healthcare with soft robotic devices: A review

**DOI:** 10.1002/SMMD.20230045

**Published:** 2024-02-23

**Authors:** Yuzhe Wang, Zhen Xie, Huishi Huang, Xinquan Liang

**Affiliations:** ^1^ Singapore Institute of Manufacturing Technology Agency for Science, Technology and Research (A*STAR) Singapore Singapore; ^2^ Advanced Remanufacturing and Technology Centre Agency for Science, Technology and Research (A*STAR) Singapore Singapore; ^3^ Department of Mechanical Engineering National University of Singapore Singapore Singapore

**Keywords:** implants, prosthetics, rehabilitations, soft robotic devices, soft sensors, surgical interventions, wearables

## Abstract

Recent advancements in soft robotics have been emerging as an exciting paradigm in engineering due to their inherent compliance, safe human interaction, and ease of adaptation with wearable electronics. Soft robotic devices have the potential to provide innovative solutions and expand the horizons of possibilities for biomedical applications by bringing robots closer to natural creatures. In this review, we survey several promising soft robot technologies, including flexible fluidic actuators, shape memory alloys, cable‐driven mechanisms, magnetically driven mechanisms, and soft sensors. Selected applications of soft robotic devices as medical devices are discussed, such as surgical intervention, soft implants, rehabilitation and assistive devices, soft robotic exosuits, and prosthetics. We focus on how soft robotics can improve the effectiveness, safety and patient experience for each use case, and highlight current research and clinical challenges, such as biocompatibility, long‐term stability, and durability. Finally, we discuss potential directions and approaches to address these challenges for soft robotic devices to move toward real clinical translations in the future.

## Graphical abstract

The use of soft actuation mechanisms in robotics has expanded the horizons of possibilities for biomedical applications and brought robots closer to humans. This review surveys several promising soft robotic technologies and presents selected applications of soft robotic devices as medical devices. Current research and clinical challenges are highlighted, and potential future directions to address these challenges for soft robotic devices to move toward real clinical translations are discussed.

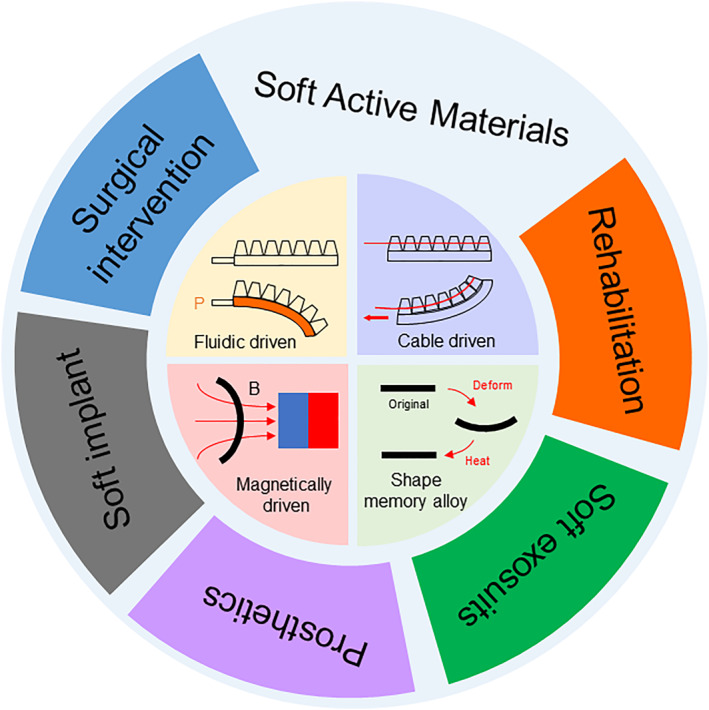

**Key points**

- Outline the recent development of soft robotic technologies that are promising for healthcare use cases.
- Selected applications of soft robotic devices as biomedical instruments are introduced.
- Highlight current research challenges and potential future directions for soft robotic devices to move toward real clinical translations.

## 1 INTRODUCTION

The healthcare sector continuously seeks innovative solutions that can improve the safety, efficiency, and overall patient outcomes of medical procedures. Robots in biomedical and healthcare applications date back a few decades and have created significant benefits across various medical specialties. To unlock the full potential of robotics for biomedical applications, it is significant for medical practitioners to stay aware of the most recent developments in this domain. Recently, the use of soft actuation mechanisms in robotics has expanded the horizons of possibilities for biomedical applications.^1^ As a rising subfield of robotics, soft robotic devices are primarily made of easily deformable materials, such as fluids, gels, and elastomers with similar elasticity and compliance of biological tissues,^2^ bringing robots closer to nature creatures and emerging as an exciting paradigm in engineering with the potential to reshape the biomedical and healthcare field. In general, soft robotic devices allow for safer clinical interactions with patients, intrinsically reduce mechanical complexity, adapt to complex working environments, and provide practical potential for medical device development and training.^3^

The applications of soft robotic devices in healthcare are multifaceted and ever‐expanding. For example, in invasive surgery and endoscopy, the intrinsic compliant nature of soft robots allows for higher flexibility and dexterity to perform tasks with gentleness in narrow in vivo spaces. For rehabilitation and assistance applications such as exosuits and rehabilitation gloves, soft robots can seamlessly conform to the human body with better comfort and drive motions more akin to nature muscles, which play a significant role in assisting individuals in regaining lost mobility and function. Soft robots can also be used as prosthetics to replace human body parts or artificial organs. Moreover, soft devices are also emerging as powerful tools in sensing physiological signals, contributing to the development of innovative diagnostic devices and systems, not only improving existing healthcare procedures but also pioneering new paradigms in personalized wearable health monitoring and implantable devices. In this review, we discuss the current biomedical applications of soft actuation mechanisms, including fluid‐driven, Shape memory alloys (SMAs), cable‐driven, and magnetically driven mechanisms. We survey the applications of soft robotic devices as medical devices for surgical intervention, soft sensing and implants, rehabilitation, soft robotic exosuits, and prosthetics.

## 2 SOFT ACTUATORS

**FIGURE 1:**
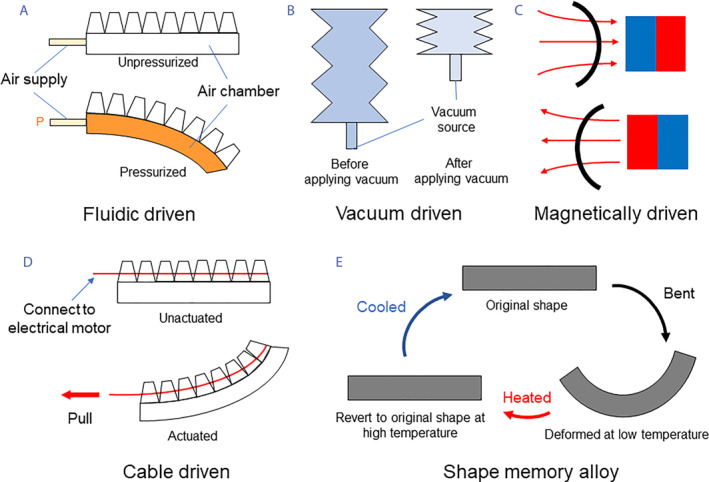
Promising soft actuators for biomedical applications include (A) fluid‐driven soft actuators, (B) vacuum‐driven soft actuators, (C) magnetically driven soft actuators, (D) cable‐driven soft actuators, and (E) shape memory alloys.

Many soft actuation technologies have been explored in the past decades to enable flexibility, compliance, and safety for biomedical applications.^4^ Among all the soft actuation technologies, there are several promising strategies that stand out, including flexible fluid‐driven mechanisms, SMAs, cable‐driven mechanisms, and magnetically driven mechanisms (Figure 1).^5^, ^6^

Flexible fluidic actuators are flexible inflatable structures driven by fluids. Typically, there are three types of flexible fluidic actuators, including hydraulic‐driven actuators,^7^, ^8^ pneumatic‐driven actuators,^9^, ^10^ and vacuum‐driven actuators,^11^, ^12^ depending on the actuation fluid (air or liquid) or type of pressure (positive pressure or negative pressure). All these actuators consist of a flexible chamber usually made of elastomeric materials (such as silicones and polyurethane), hydrogels, plastics, and cloth. These flexible chambers can bend, twist, contract or extend when subjected to changes in the internal pressure, depending on their designs and configurations such as pneumatic bellows, accordion‐like structures, origami structures or more complex designs.

Flexible fluidic actuators are safe, soft, and compliant to interact with biological structures and tissues, and they can reduce the risk of tissue damage or discomfort during treatment. These properties make them suitable for various medical and healthcare applications, such as wearable rehabilitation devices and soft robotic exosuits. The actuation is also similar to natural muscle movement biological systems, making them well‐suited for replicating muscle contraction and extension in prosthetics. Despite these ideal properties, flexible fluidic actuators also come with several issues and challenges. For example, they may not provide the same level of precision as conventional actuation methods (e.g., electric motors). The compressibility and fluid dynamics of air or liquids may lead to hysteresis and nonlinearities in motion, which imposes challenges in achieving high precision. Another challenge of implementing flexible fluidic actuators in biomedical applications is the scalability which involves manufacturing challenges for miniaturization of the flexible chambers, and the limited commercial availability of miniature valves, micropumps, and thin pipes.

SMAs exhibit “shape memory behaviors” which allows them to restore a predefined shape when subjected to the appropriate thermal conditions.^13^ The two most widely used SMAs are copper‐aluminum‐nickel and nickel‐titanium. To generate reversible actuation, SMA is first deformed into the desired shape at a temperature below its transformation temperature (at its martensite crystallographic phase). When the alloy is subsequently heated to or above its transformation temperature (at austenite crystallographic phase), it returns to its original shape, exerting mechanical forces or displacements. In the past few decades, SMA‐based soft actuators have been extensively studied due to their excellent properties, including lightweight, quiet operation, high‐energy density, compactness, high corrosion resistance, and biocompatibility.^14^, ^15^, ^16^ This type of soft actuator can exert localized forces depending on specific shapes and dimensions, with advantages in small scales. As many SMAs are biocompatible, they are safe for use in contact with biological tissues, making them suitable for medical use. However, SMAs also have several limitations, including temperature sensitivity, limited actuation speed, limited displacement, and complex thermomechanical behaviours. In addition, their force output decreases when the actuator approaches the end of its stroke, and the repeating transition between the martensitic and austenitic phases may lead to material fatigue and reduce their lifespan. Despite these limitations, the unique properties of SMAs are still valuable to provide innovative solutions for biomedical applications such as rehabilitation and assistive devices.

Cable‐driven soft actuators produce motion and force using cable systems. This type of actuator typically consists of a deformable structure made of compliant materials, such as silicone elastomers, natural rubber, polyurethanes, or plastics. Cables made of Nylon, rubber, or metals are routed through or attached to various points of the deformable structure and anchored to external tensioning systems such as motors and pulleys. By adjusting the tension in the cables, the cables can exert tensional forces and create deformations in the flexible structure.^17^, ^18^, ^19^ Compared to other soft actuation methods that are challenging to achieve the same level of precision as conventional actuation methods, the cable‐driven approach allows for more precise control of the shape and movement of flexible structures since the cable movement is controlled by electrical motors outside the actuator body. In addition, cable‐driven soft actuators can also exert higher force, making them suitable for soft exoskeletons, wearable assistive devices, and medical rehabilitation tools. However, achieving a complex multi‐degree‐of‐freedom motion in cable‐driven systems is still a major challenge. Multiple cables routed through the soft structure may interfere with each other, leading to unintended deformations or conflicts in the actuation process. Cable routing and tensioning require careful consideration to avoid such issues. In addition, cables can only exert tensile forces. The actuator will become unstable or out of control when the cables lose their tension.

Magnetically responsive materials, such as carbonyl iron, iron oxide, soft ferrite, iron‐silicon alloys, and iron‐nickel alloys, can respond to external magnetic field to perform deformations due to programmed magnetic dipoles.^20^ Soft actuators made of magnetically responsive materials can be remotely controlled to achieve locomotion by external magnetic fields instead of carrying a power supply and control system, making them an effective strategy for miniaturized, lightweight, and untethered soft robotic devices.^21^, ^22^, ^23^ Magnetically driven soft actuators can be integrated with functional surfaces and structures such as reconfigurable functional surfaces, origami structures, and metamaterial structures to achieve complex and dexterous motion. Magnetic soft robots also exhibit good biocompatibility, making them attractive in applications such as targeted drug delivery and minimally invasive robotic surgery for previously inaccessible lesions, providing a new method for in vivo therapy and surgery with remote control outside patient's body. Currently, multi‐physics actuation is still a challenge for magnetically driven soft actuators to couple with other actuation mechanisms. There is also an urgent demand for the development of self‐healing soft magnetic materials which can enhance environmental adaptability and mechanical durability.

Designing actuators with soft materials presents significant challenges due to increased complexity and degrees of freedom within soft robotic systems. In the early stages of soft robotics research, researchers often use try‐and‐error approaches based on their intuitions and experiences to design soft actuators, which can be inefficient and lack of established guidelines. In response, research efforts have progressively shifted towards more comprehensive design approaches. One such approach involves optimization‐based design methods which integrate a unified mathematical representation of state variables and physical laws governing soft materials.^24^ These methods leverage powerful simulators, optimization algorithms, and high‐performance computing to enable rapid design iterations for both materials and machines. This framework holds the potential for significant advancements in the optimization of soft robotic systems. Another design framework is based on the evolutionary design approach, which combines elements more akin to natural evolution or evolutionary algorithms.^25^ Starting from a general concept of design option, several different configurations, that can be referred to as “phenotypes,” are evolving through their own evolution pathways by adding the minimum number of details in each iteration and ensuring their cross‐interactions to achieve the targets iteratively and incrementally through design‐test cycles. In retrospect, this iterative design approach is reminiscent of evolution by natural selection, where the complexity of the functions and the vastness of the design space preclude existing mathematical frameworks. The establishment of these diverse design approaches is an important step towards enhancing the performance of soft actuators for targeted applications. Moreover, they serve as efficient tools for designing soft actuators and robots featuring more complex geometries and functionalities.

## 3 SOFT SENSORS AND IMPLANTS

**FIGURE 2:**
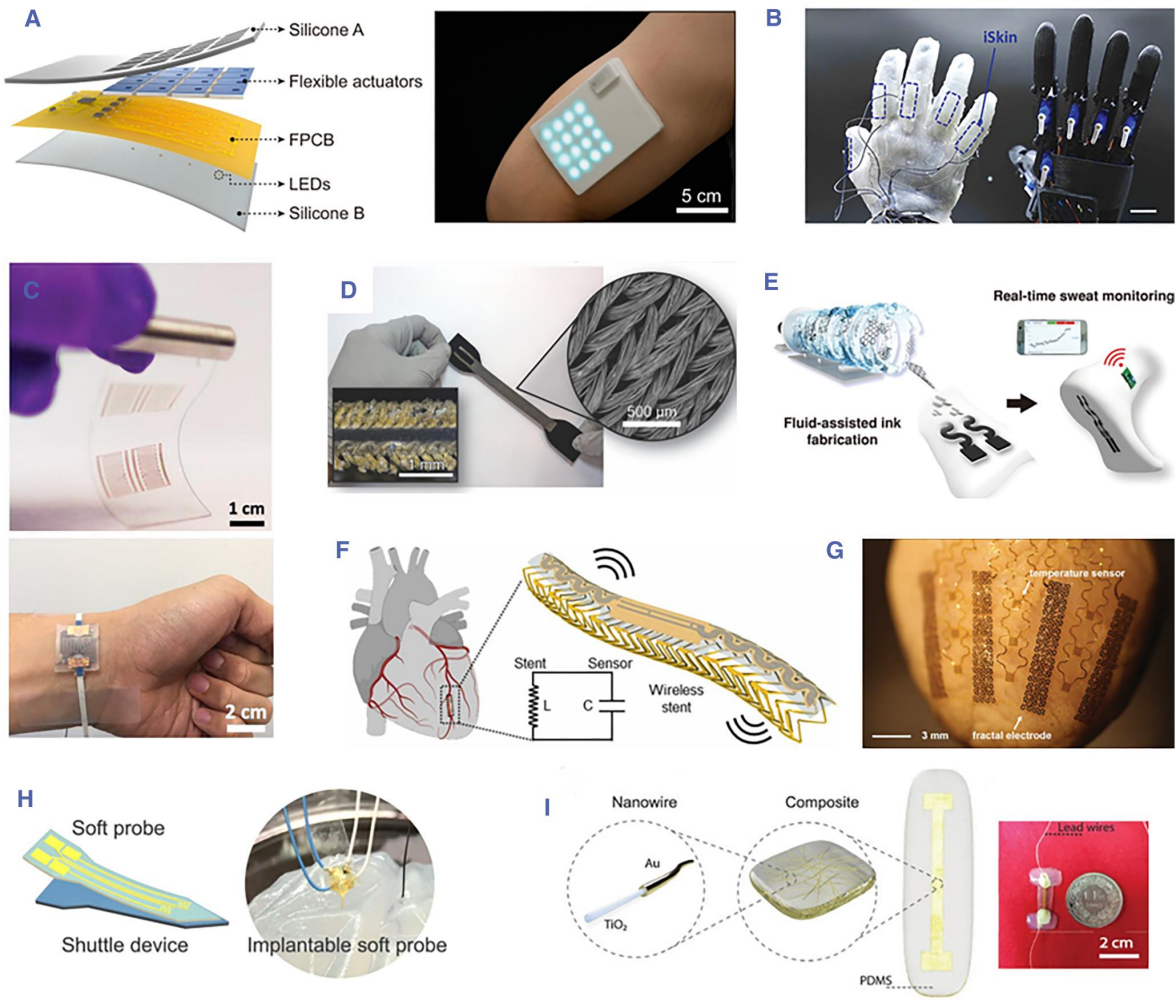
(A) Flexible self‐sensing actuator with electronic skin, providing an integrated function in both tactile sensing and haptic feedback. Reproduced under terms of the CC‐BY license.^28^ Copyright 2022, The Authors, published by the American Association for the Advancement of Science. (B) Hydrogel‐based ionic skin (iSkin) capable of strain sensing. Reproduced with permission.^29^ Copyright 2021, John Wiley & Sons. (C) Inkjet‐printed soft resistive pressure sensor patch. Reproduced with permission.^30^ Copyright 2020, John Wiley & Sons. (D) Customizable silicone‐textile composite capacitive strain sensors. Reproduced with permission.^31^ Copyright 2017, John Wiley & Sons. (E) Eco‐Gr ink for smart socks with Na+ sensor and reference electrodes. Reproduced with permission.^32^ Copyright 2022, American Chemical Society. (F) Implantable soft platforms with printed nanomaterial‐based arterial stiffness sensors. Reproduced with permission.^33^ Copyright 2022, Elsevier. (G) Implantable soft device incorporated into a Langendorff‐perfused rabbit heart. Reproduced with permission.^34^ Copyright 2015, John Wiley and Sons. (H) Flexible and implantable polyimide aptamer‐field‐effect transistor biosensors. Reproduced with permission.^35^ Copyright 2022, American Chemical Society. (I) Fully implantable soft strain sensor for continuous heart‐volume monitoring. Reproduced with permission.^36^ Copyright 2020, John Wiley & Sons.

With the advancements in sensor materials in the past few decades, stretchable and soft electronic devices have been emerging rapidly and envisioned as the future of next‐generation personalized wearable health monitoring and implantable devices.^26^ Sensors play a crucial role in identifying and quantifying changes in vital signs, providing a holistic view of patients' health conditions. Given the soft nature of human skin and tissues, sensors must exhibit flexibility and stretchability to seamlessly integrate with devices or be directly applied to the human body. To meet these demands, various types of flexible and stretchable sensors have been developed,^27^ including physical sensors, chemical sensors, and biological sensors, based on the sensing mechanisms. Physical sensors measure various physical parameters and convert them into readable signals, often involving changes in electrical properties, resistance, magnetic fields, triboelectric properties, or light intensity. Chemical sensors detect and quantify specific chemical analytes, such as ions, molecules, or compounds, in biological samples through active electrodes tailored to respond to specific chemical species. Biological sensors harness biological components such as enzymes, antibodies, nucleic acids, and whole cells to detect and interact with target analytes. In this chapter, we provide an overview of three different types of soft sensors: elastomer‐polymer‐based sensors, textile‐based wearable sensors, and implantable sensors, based on the aforementioned mechanisms (Figure 2).

### 3.1 Elastomer‐polymer‐based sensors

Elastomer‐polymer‐based sensors typically incorporate active filler materials into intrinsically stretchable elastomeric polymers. This type of sensors can effectively cater to the sensing requirements of highly compliant soft robotic devices as the mechanical properties of the sensors, such as stiffness, can be made significantly lower than those of the robot body to ensure that the sensors do not interfere with the behavior of the soft robotic devices and remain functional even under significant strains. These sensors can address sensing challenges in soft robotics, aiding in state perception and system identification for healthcare procedures by providing feedback on parameters such as deformation and forces.^37^, ^38^ Active filler materials such as hydrogels, metals, carbon‐based materials, and conductive polymers have been successfully employed in combination with various elastomeric polymer substrates, including polyethylene terephthalate, polydimethylsiloxane (PDMS), and silicone rubbers.

Hydrogels are excellent conductors due to their constant resistivity to remain independent of deformation. This property is especially valuable under large strains, making hydrogels an ideal choice as active filler materials for resistive soft sensors. For example, a facile “stick‐on” method is proposed to bond a layer of silanes‐modified hydrogel onto a silicone‐rubber and integrate it into a soft pneumatic actuator.^39^ This sensor effectively measures the bending angles of the actuator with minimal constraint. In another work, a sensor was designed for multi‐modal perception of soft fingers to monitor bending, twisting and external forces with high sensitivity.^40^ To address issues like water evaporation and low stickiness, the hydrogel is encased in Very‐High‐Bond tape. How the placement of the soft sensor minimally affects the dynamics of the pneumatic fingers while ensuring optimal strain capture during actuation is also investigated.

Ionic liquid or liquid metal are also common materials for resistive soft sensors.^41^ When injected into microfluidic channels and sealed, these channels can deform together with the substrate under external loads, leading to changes in resistance. This change can be calibrated to measure related loads. For example, stretchable fluorinated copolymer ionogels with enriched ionic liquids can be used for human‐motion detection. The skin‐inspired wearable sensors with waterproof performance are suitable for the real‐time detection of physiological human activities in complex and extreme environments.^42^ Liquid metal‐based pressure sensors can achieve high sensitivity with a robust interconnection structure. They can be used as fingertip pressure sensors for wrist pulse monitoring, which is commonly one of the diagnoses in traditional East Asian medicine.^43^

### 3.2 Textile‐based wearable sensors

Textile‐based wearable sensors have been developed for direct wearability to enable real‐time, continuously, and non‐invasive detection of human physiological signs (heart rate, respiratory rate, body temperature, etc.) or biomarkers in bodily fluids, facilitating a comprehensive analysis of an individual's health.^44^ Human skin motion involves a combination of stretching and wrinkling, with the capacity to stretch elastically up to 15%. However, during daily activities, skin deformation can reach up to 100%. To ensure these sensors maintain conformal contact with the skin surface while allowing for human movement, the sensors must possess low modulus and high flexibility.^45^ Common materials used to fabricate these sensors include fibers, yarns, and fabrics. These attachable sensors can be incorporated with textiles that are worn on the skin to meet user comfort, breathability, biocompatibility, and lightweight.

For example, a multifunctional tactile sensor capable of mimicking human skin mechanoreceptors by integrating triboelectric and piezoresistive materials has been developed.^46^ When applied to the wrist surface, it offers real‐time monitoring of heart rate by detecting pulse rate fluctuations. Textile‐based temperature sensors usually rely on changes in thermal resistance to measure temperature variations. A silk‐based temperature sensor demonstrates remarkable sensing performance, with a high sensitivity of 0.81% resistance change per degree Celsius.^47^ This sensor exhibits minimal response to external pressure stimuli, ensuring accurate readings even under varying conditions. It also shows cyclic and stable signals in response to cyclic exhalation stimuli.

Some soft sensors are capable of real‐time monitoring of biological fluids, such as sweat, tear, and saliva. These sensors offer insights into various chemical compositions and provide comprehensive understandings of individual health status. Textile‐based sweat sensors primarily utilize electrochemical methods to respond to metabolites in sweat.^48^ Enzymes anchored to the working electrodes play a crucial role in this process. Wearable sweat sensor socks can be integrated with printed serpentine‐patterned stretchable graphene electrodes.^32^ These sensors can monitor the sodium ion (Na+) concentration in the sweat of individuals during indoor stationary cycling exercises based on the printed graphene conductor. In another work,^49^ hydrophilic Polyacrylonitrile/Polyvinylpyrrolidone/Valinomycin nanofibers (PPVN) yarns are woven with hydrophobic polyester yarns to form a plain fabric so that the sweat can be completely confined in the PPVN yarns. This fabric acts as a specific receptor for potassium ions (K+), and valinomycin selectively detects K+ and generates the corresponding electrical potentials.

Besides the primary focus on monitoring changes in the physiological signs of the human body, sensors can also be strategically attached to different body parts for muscle movements or posture tracking. For example, a textile‐silicone capacitive sensor is used for human articulation detection.^31^ This sensor exhibits capacitance changes when the electrode area and dielectric thickness change geometry in response to applied strain, making it suitable for hand motion tracking when integrated into a glove. The multifunctional tactile sensor presented in Ref. 46 can also accurately detect muscle movements during speech and thereby recognize the voice patterns when attached to the throat.

### 3.3 Soft implants

Soft implants are designed to monitor neural signals and stimulate neurons for applications in prosthetics, neural signal recording, and neuromodulation. These devices must be chronically implantable, biocompatible, biostable, and compact enough to measure signals from focused regions of the brain or nerves. Thus, inert materials that do not induce immune responses are required to ensure biocompatibility and bio stability of soft implants. Common materials used in these implants include silicones, hydrogels, parylene, and polyimide. Combined bio‐compatible multifunctional materials can achieve higher dexterity, larger variability of movements, and accurate sensing capabilities. Combining tissue engineering approaches and bio‐derived structural materials with soft robotic devices for implants and surgical tools is a promising research direction which has been receiving attention. Currently, soft sensing materials which are used in soft implants can be categorized into two primary categories based on their target areas: central nervous system (CNS) electrodes and peripheral nervous system (PNS) electrodes.^50^

CNS electrodes are designed for the precise measurement of evoked potentials originating from neurons within the CNS, which comprises the brain and spinal cord. An essential requirement for these electrodes is to establish a seamless and conformal contact with the often intricate and folded tissue of the CNS, ensuring accurate and reliable acquisition of signals from the CNS. For example, a device has been designed to integrate electrodes with the entire 3D surface of the heart by featuring an array of eight strategically placed electrodes around the circumference of the heart, which offers unique operational capabilities for low‐power defibrillation and ensures accurate sensing of intrinsic electrophysiological activities.^34^ In addition, there have been developments in highly stretchable electrodes on porous PDMS substrates.^51^ An electroplated nickel anchor bonds commercial electronic components to elastomers through soldering techniques. This electrode successfully measures evoked potentials in rats induced by limb stimulation.

PNS electrode comprises a complex network of nerve fibers. Implantable electrodes targeting the PNS have been proven effective in the form of cuff electrodes. These specialized electrodes encircle and envelop nerve surfaces within the PNS. They are designed for chronic implantation with a primary focus on minimizing any potential harm to the nerves while ensuring stable and reliable electrical connections for a range of applications. For example, polyimide nerve cuff electrodes have been demonstrated with controllable drug loading/release functions to facilitate stable recording of peripheral nerve signals and stimulation while minimizing inflammation.^52^ Another self‐closed parylene cuff electrode developed in Ref. 53 has been characterized for peripheral nerve recording on the rat sciatic nerve, verifying its capability to record neural activities effectively.

## 4 SOFT ROBOTS FOR SURGICAL INTERVENTIONS

The mechanical flexibility and inherent compliance of soft robotic devices allow for safer and more effective integration within interventional systems across various clinical fields. Nowadays, minimally invasive surgery (MIS) has gained popularity for many abdominal procedures compared to the traditional open surgical approach due to reduced postoperative discomfort, faster recovery, shorter hospital stays, and improved esthetic outcomes.^54^ Based on the MIS, one novel variation gaining traction is the natural orifice transluminal endoscopic surgery (NOTES), which circumvents the need for external incisions. An endoscope may be introduced through a natural external orifice, such as the mouth, anus, vagina, or urethra to thereby visualize various cavities or further create internal passages to allow access to the peritoneal cavity and various viscera.^55^

Both MIS and NOTES procedures usually rely on a cone‐shaped workspace originating at the incision or natural orifice. However, the restricted range of motion can pose challenges when navigating around multiple organs to find the route to the target site, and highly adaptable and flexible tools can effectively help surgeons to perform these intricate procedures. Thus, it would be significant to have small, flexible, and strong manipulators which can reach difficult‐to‐access surgical sites via curved pathways and complete the surgical task with dexterity.^56^

In this chapter, we focus on soft continuum robots in surgical intervention, which are promising candidates for tackling the abovementioned challenges. Soft continuum robots can be further divided into two categories based on their sizes and applications: soft endoscopes, typically at millimeter or centimeter scales, and soft catheters, operating at sub‐millimeter scales. Offering virtually infinite degrees of freedom, these soft robots can adapt to various surgical demands by extending, contracting, and bending. Made of bio‐compatible materials such as silicones, elastomers, or SMAs, soft continuous robots are safe to be used inside the human body with less risk of damaging delicate soft tissues. Furthermore, biocompatible soft actuators can be made of patients' biological cells or DNA‐based molecular machines, which allows the surgical tools to be accepted by the immune system, paving the way for biohybrid soft robots for surgery.

### 4.1 Soft endoscopes and manipulators

Soft endoscopes are used for abdominal MIS and certain NOTES procedures such as transesophageal and transcolonic routes. Common techniques in the surgery include navigation through narrow and complex pathways to reach the desired target, stiffness control to stable itself when reaching the goal, and the subsequent manipulation in various ways including ablation, and biopsy. Cable driven actuators via a pair of antagonistic tendons, pneumatic/hydraulic‐driven actuators, and concentric tubes made of pre‐curved elastic tubes nested in each other are the most common actuators for soft endoscopes.

Flexibility is a crucial attribute for soft endoscopes and manipulators in the aspects of shapes varieties, stiffness ranges, and force adequality. It is necessary for soft medical robots to gain adaptability to modulate their flexibility over a wide range to execute diverse tasks effectively.^57^ A passive mode characterized by high compliance and low stiffness is essential. This mode allows them to navigate through restricted spaces, deform, and interact delicately with soft organs. Conversely, an active mode provides low compliance and high stiffness to maintain specific positions, retract tissues, remove tumors, or suture areas upon reaching the targeted region. Thus, the capability to switch between passive and active states is the focus of soft endoscope and manipulator designs. Furthermore, certain output torques are also crucial based on the above requirements to apply forces and adjust their load‐carrying capacity.

#### 4.1.1 Stiffness control

**FIGURE 3:**
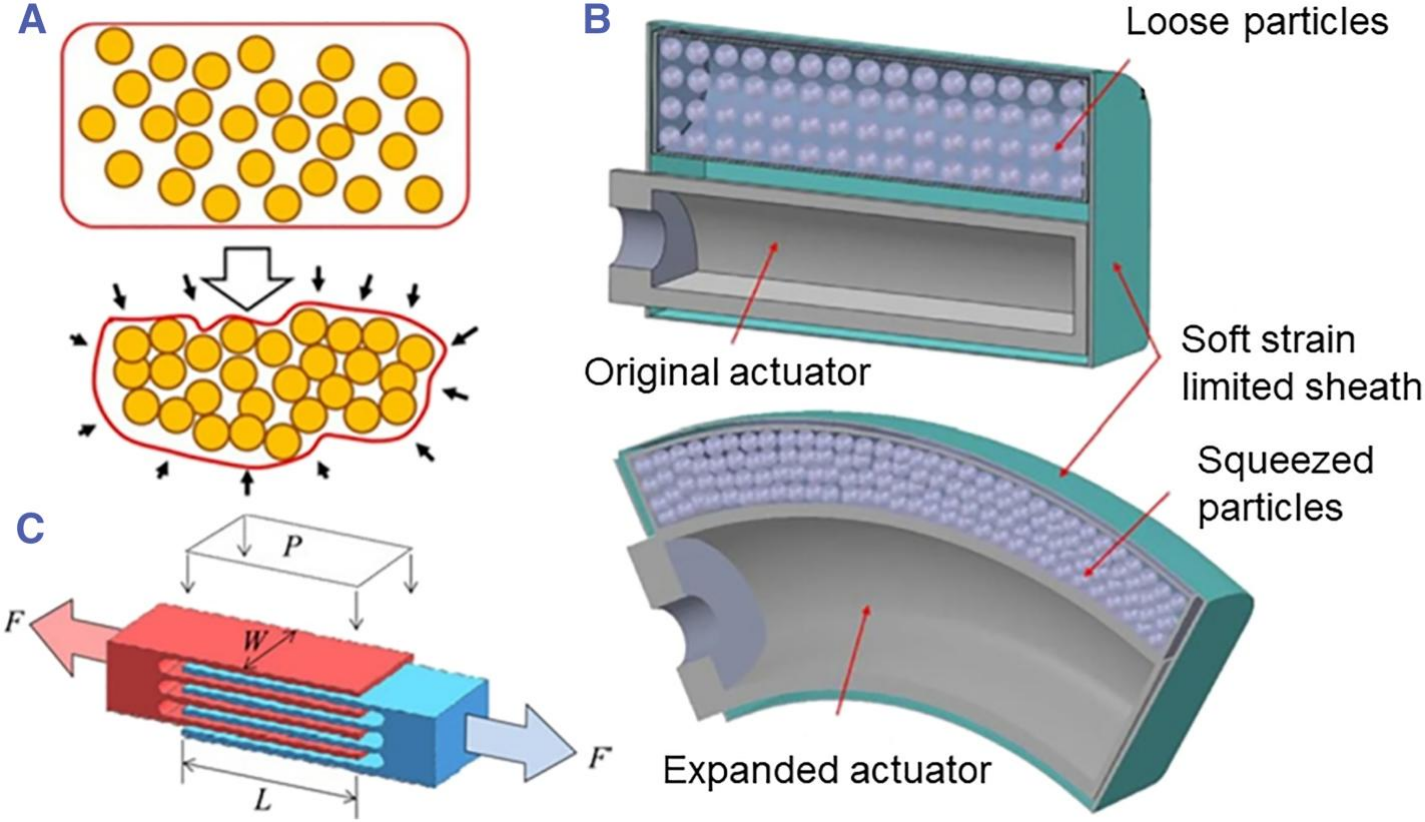
Stiffness control mechanism. (A) Active jamming. (B) Passive jamming. (C) layer jamming. Reproduced with permission.^57^ Copyright 2020, John Wiley and Sons.

Variable stiffness can be achieved by three different types of mechanisms: jamming‐based methods, intrinsic stiffness‐tunable materials, and antagonistic arrangement. Jamming is a mechanism in which the change in stiffness is governed by the slip at the interfaces between the granular‐based materials such as coffee powder, sand, and plastic particles, or layer‐based materials such as silicone sheets and rubber sheets of plastic film layers. These designs utilize loosely packed granular materials or layered materials inside a flexible capsule. When subjected to vacuum, the granules or layers are squeezed to increase the stiffness and provide support. By controlling the level of vacuum inside the capsule, the friction between granules or layers can be adjusted, allowing for the adjustable and reversible stiffness control (Figure 3). The range of stiffness is often illustrated as tensile, compression and bending stiffness change ratios for jamming systems in soft robotics. For example, granular jamming can tune the compressive (up to 40× original stiffness) and bending stiffnesses (10× original stiffness) effectively. However, vacuum‐packed granules can only achieve 5× original stiffness when resist tensile forces, making it unsuitable for constraining tensile strains. Traditional layer jamming can only be used to tune the bending stiffness (50× to 300× original stiffness) since it does not have any in‐plane degrees of freedom. The recent development of stretchable layer jamming can also tune tensile stiffness (50× to 100× original stiffness) as individual layers are not connected to all sides of the device, and the layers can slide past each other when pulled.^58^

Flexible fluidic actuators can be combined with granular jamming mechanism to design a modular soft manipulator for MIS.^59^ For layer jamming, the design of a snake‐like manipulator with layer jamming scales is demonstrated.^60^ The hollow shape manipulator can change to a rigid state after reaching a threshold and can be used as a guide tube to deliver other MIS tools during surgery. Another similar jamming mechanism utilizing a silicone sponge is also proposed.^61^ The variable‐stiffness device is operated by compressing the open cells inside the sponge to store elastic energy within the distorted elastomer. The stored energy enables the sponge to quickly return to its initial configuration upon the release of the jamming force.

Tunable‐stiffness materials can transfer between their liquid and solid phases when subjected to temperature changes. For example, low‐melting‐point alloy (LMPA) can achieve a large stiffness variation ratio, and a novel gastrointestinal endoscope to accomplish both LMPA‐based variable stiffness and multi‐DOF actuation is developed based on this mechanism. The lateral and flexural stiffness change ratios are 13.15 and 477.05, respectively.^62^ This manipulator integrates three hydraulic‐driven LMPA chambers and three lumen chambers dedicated to water circulation. A long balloon wraps the multicavity silicone hose, which facilitates the flow of water to regulate the temperature of the LMPA, possessing a melting point of 29.78°C, thereby enabling cooling or heating as required. In addition, mechanical structures of antagonistic arrangement can also achieve variable stiffness. A hyper‐redundant manipulator with a unique variable neutral‐line mechanism is designed to change the stiffness by varying the tension of the asymmetric tendons connected to the manipulator.^63^

#### 4.1.2 Navigation and tissue manipulation

Navigating the manipulator to the target position via a complex environment and executing tissue manipulation such as ablation or biopsy are the two fundamental steps in the surgery process. Most of the surgery devices are designed for a single step. The navigation is completed by the soft endoscopes, while the manipulation is accomplished by additional functional tools inserted and guided via the central channel of the soft manipulator. Recently, there are some designs aiming to complete these two steps with a single integrated system, which pose greater challenges but higher demands.

Navigation is one of the key research areas for soft endoscopy. Inspired by the elephant nose, researchers have created a cable‐driven soft endoscopic probe for cardiac ablation.^64^ Eight fiber cables with different configurations enable different moving patterns such as blending, S shape, wriggling and hybrid modes to navigate in the pericardium. Ablation tools and a micro camera with lights were embedded in the central channel of the probe. In another study, planning of a soft‐rigid hybrid arm for the transrectal biopsy of the prostate is investigated.^65^ The procedure consists of moving the ultrasound probe connected via a compliant soft part to the rigid robot arm near the prostate through the rectum and aligning it with identified lesions in the prostate.

Efforts are made to integrate the navigation and tissue manipulation processes into one single device. For example, an MR‐safe soft robotic system for MRI‐guided transoral laser microsurgery is proposed.^66^ This soft robot integrates the active bending section for coarse navigation, and a distal manipulator tailored for precise adjustments. This laser manipulator is constructed using a combination of flexible and rigid materials. It operates on a micro‐scale fluid flow with <0.004 mL volume, which facilitates precise and consistent control of laser direction. Another research introduced a soft suction‐based gripper mounted on the end effector of a soft endoscope for endoluminal manipulation. This device can generate forces, provide tissue retraction, and enable cutting.^67^

### 4.2 Guild wire and catheters insertion

**FIGURE 4:**
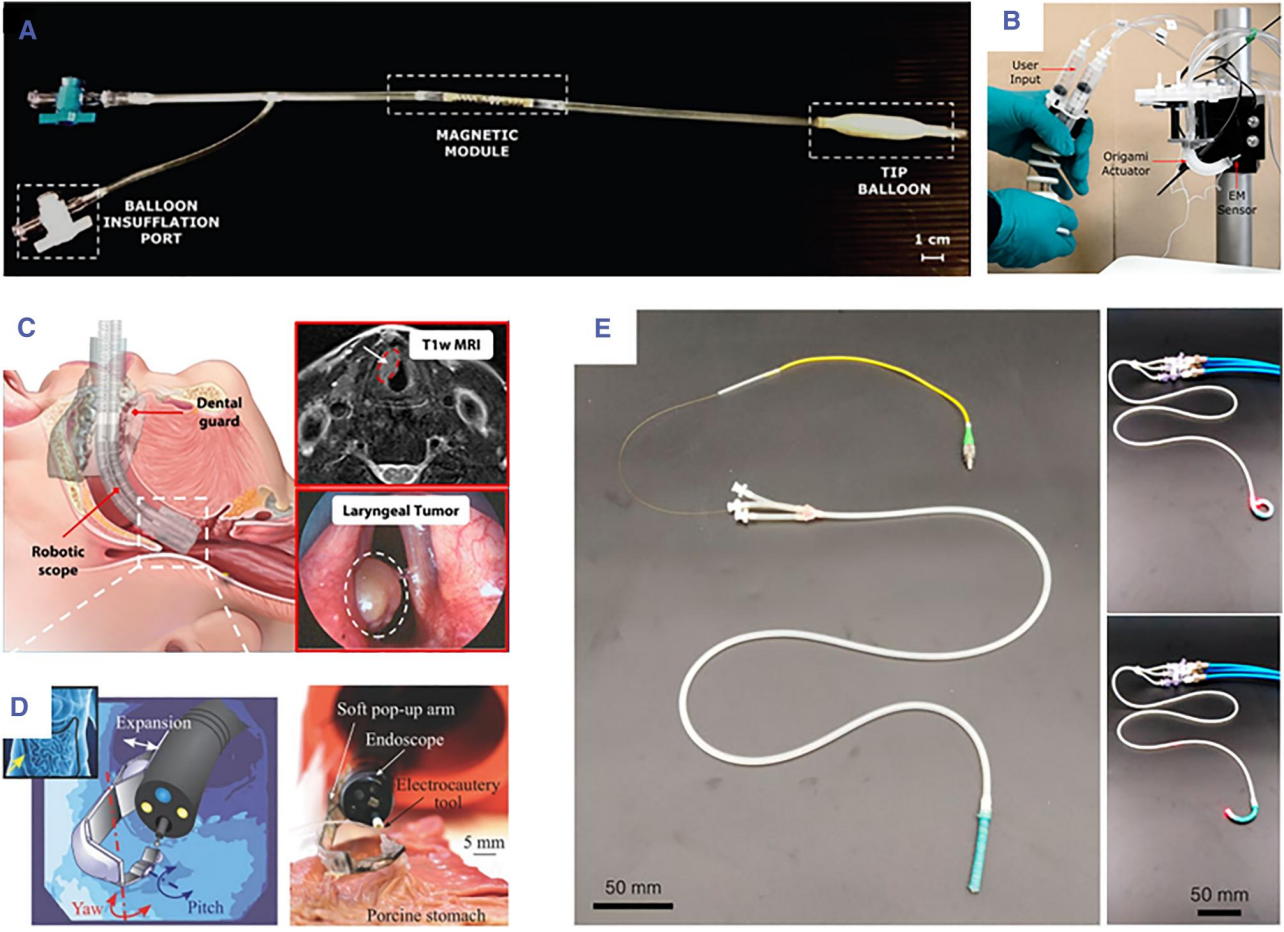
(A) Intravascular retrieval catheter prototype. Reproduced under terms of the CC‐BY license.^68^ Copyright 2018, The Authors, published by John Wiley and Sons. (B) Origami‐based soft robotic actuator for upper gastrointestinal endoscopic applications. Reproduced under terms of the CC‐BY license.^69^ Copyright 2021, The Authors, published by Frontiers. (C) Soft robotic manipulator for intraoperative MRI‐guided transoral laser microsurgery. Reproduced with permission.^66^ Copyright 2021, The Authors, published by American Association for the Advancement of Science. (D) A soft pop‐up arm performs tissue counter‐traction during an ex‐vivo test on a porcine stomach. Reproduced with permission.^70^ Copyright 2017, John Wiley and Sons. (E) Soft pneumatic two‐degree‐of‐freedom steerable catheter. Reproduced under terms of the CC‐BY license.^71^ Copyright 2021, The Authors, published by Frontiers.

Minimally invasive vascular surgery usually employs catheters and guidewires in submillimetre scale to perform injections, drug delivery/recovery, and placing stents for typical treatment such as angioplasties, aneurysms, and embolization (Figure 4).^68^ Challenges arise due to the catheter's length, shape, and friction against the vascular walls as the clinician advances the catheter deeper into a patient.^56^ Magnetic actuation is one of the promising technologies in this context. This mechanism involves manipulating the catheter tip by altering the intensity and orientation of the magnetic field, enabling wireless and flexible control at the sub‐millimeter scale. The large insertion depth can address challenges such as undesired trauma, reducing radiation exposure, and access to tortuous anatomy.

Magnetic actuation can be achieved by mounting a permanent magnet on the catheter under an external magnetic field generated by an electromagnetic device or magnetic resonance imaging (MRI).^72^ Permanent magnets are pre‐magnetized and generate magnetic fields without an external power supply. A procedure that harnesses magnetic manipulation to direct a steerable guidewire under the guidance of ultrasound imaging is detailed.^73^ The constructed system integrates three electromagnets and an ultrasound probe into a parallel mechanism, together with a motorized feeder, realizing large‐workspace magnetic field generation and ultrasound feedback to actuate a guidewire with a permanent magnet. In MRI‐guided procedures, a balance needs to be achieved between the magnetic field required for imaging and the magnetic field used to actuate the catheter. Additional magnetic fields can be generated using the current‐carrying coils embedded in the catheter to produce magnetic fields that interact with the MRI's main magnetic field, allowing the catheter to be steered and controlled. Design optimization of an MRI actuated steerable catheter for atrial fibrillation ablation in the left atrium is presented.^74^ Material selection and coil actuator optimization are performed, and the final design is validated through simulation, demonstrating successful reachability and contact force.

## 5 SOFT ROBOTS FOR REHABILITATION AND ASSISTIVE DEVICES

**FIGURE 5:**
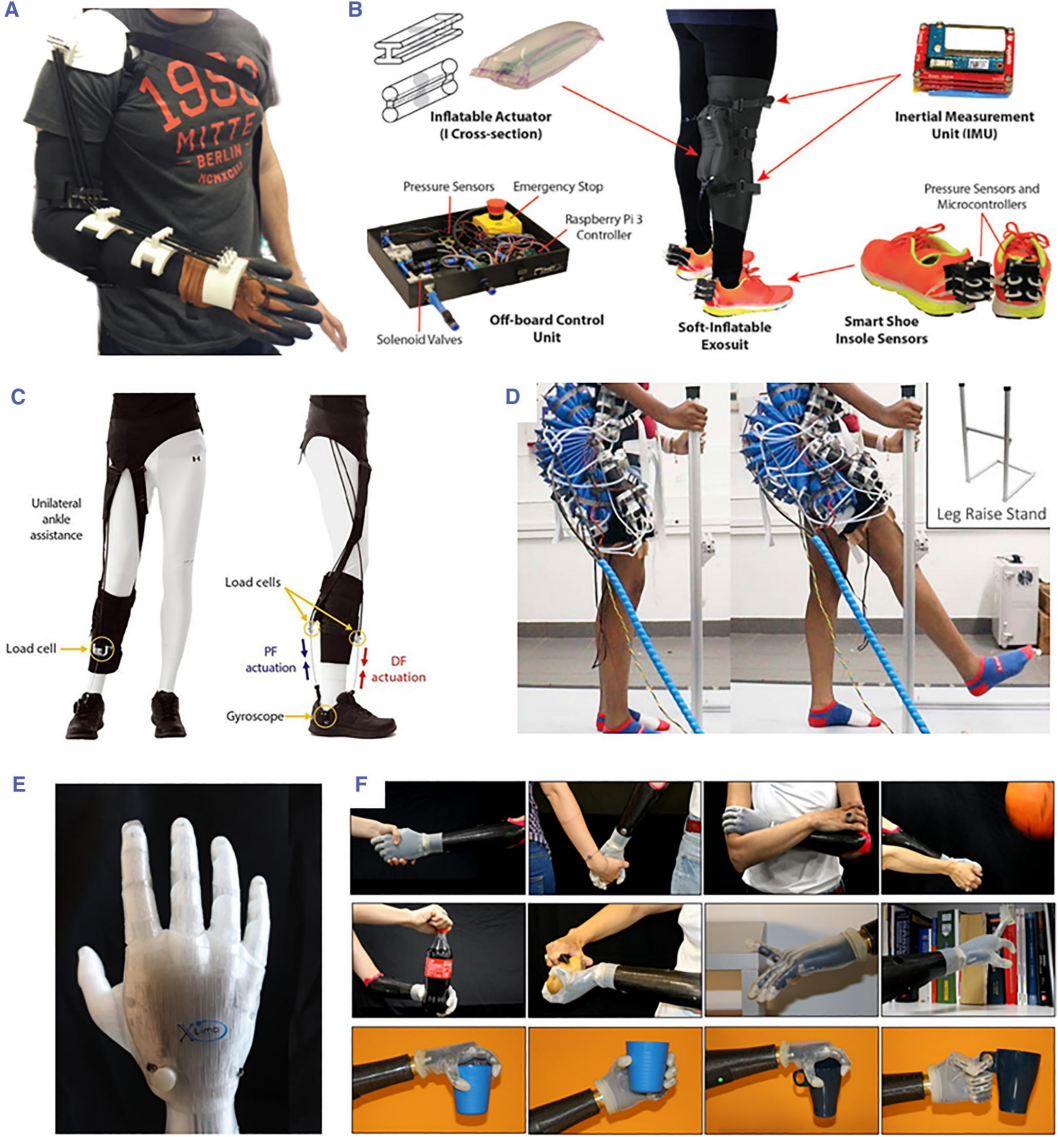
(A) Wearable exoskeleton for elbow medical rehabilitation with shape memory alloy actuators. Reproduced under terms of the CC‐BY license.^77^ Copyright 2017, The Authors, published by Hindawi. (B) System Components for soft‐inflatable exosuits. Reproduced under terms of the CC‐BY license.^78^ Copyright 2018, The Authors, published by Frontiers. (C) Soft robotic exosuit to assist the paretic ankle's gait functions after stroke. Reproduced with permission.^79^ Copyright 2017, The Authors, published by American Association for the Advancement of Science. (D) Wearable soft robotic exoskeleton for hip flexion rehabilitation. Reproduced under terms of the CC‐BY license.^80^ Copyright 2022, The Authors, published by Frontiers. (E) 3D‐printed soft robotic prosthetic hand with multi‐articulating capabilities. Reproduced under terms of the CC‐BY license.^81^ Copyright 2020, The Authors, published by PLOS. (F) SoftHand Pro system. Reproduced under terms of the CC‐BY license.^82^ Copyright 2021, The Authors, published by Springer Nature.

Soft robotics has become pivotal in the development of rehabilitation and assistive devices. The inherent compliance of soft robots addresses safety concerns, making them ideal for close human interaction. For example, the inherent softness of the materials allows prosthetic devices to mimic human skin, giving prosthetics a lifelike quality for assisting individuals with disabilities. Research has primarily focused on prosthetic hands since soft robotic devices can perform dexterous and complex movements to mimic hands. Tendon‐driven mechanisms are favored in the soft robotic research in prosthetics, ensuring precise control with substantial force and torque output. For wearable rehabilitation devices, most of them caters to joint flexibility in wrist, hand, elbow, shoulder, and gait rehabilitation. These devices use soft materials such as silicone elastomers, fabrics, and 3D‐printed materials. Pneumatic actuation is the primary mechanism, although alternatives such as SMA find applications in specific cases. Beyond prosthetics and wearables, soft robots are also utilized in various assistive devices. Wearable soft robotic limbs aid individuals in performing basic tasks, both as attachments to human^75^ and standalone devices for daily activities.^76^ Soft robotics has significantly broadened research horizons in rehabilitation and assistive devices, emerging as a key area of exploration (Figure 5).

### 5.1 Prosthetics

Soft prosthetics have gained widespread acceptance and utilization in the field of medical technology owing to their lightweight nature, ease of manufacturing, and compact size, making them popular choices among patients and healthcare professionals. Recently, hand prosthetics using monolithic 3D‐printed materials have been developed, comprising cable‐driven actuation systems, synergy‐based thumb motion, and flexure joints.^81^ Similarly, a novel monolithic soft robotic thumb that can achieve flexion/extension and abduction/adduction for an anthropomorphic and transradial prosthetic hand is proposed.^83^ The advantages of this soft under‐actuation mechanism include better performance in precision grasps, power consumption, and space consumption with one actuator focusing only on grasping versatility. An economical prosthesis (PrHand) is created using soft robotic technologies and innovative joints employing compliant mechanisms using Anthropomorphic Hand Assessment Protocol (AHAP) and Activities Measure for Upper Limb Amputees (AM‐ULA) protocols.^84^ The AHAP protocol evaluates eight different grips, considering variables such as grasping, maintaining, and grasping ability score. The second test, AM‐ULA, assesses the prosthesis's performance in 23 activities of daily living.^85^ The results show that prosthetic jamming terminal device (PJTD) has benefits over existing terminal devices because of the capability to firmly grasp tools though users experienced tiredness when using the PJTD. This work concludes that the pneumatic technology necessary for operating the PJTD is presently too bulky and heavy to allow for portable and wireless operation in a compact form.

### 5.2 Wearable rehabilitation devices

Many wearable rehabilitation devices are designed and developed from soft materials and soft actuation mechanisms. Major wearable rehabilitation devices using soft robotic technologies include soft robotic gloves and soft robotic exosuits.^86^, ^87^, ^88^ Research on other wearable rehabilitation devices, such as elbow sleeves^77^, ^89^ and hip flexion exoskeleton,^80^ are also receiving attention. Soft robotic gloves are mainly used for post‐stroke rehabilitation. Stroke patients may suffer from stiff fingers and cannot perform proper grasping in daily activities. Soft robotic gloves can assist in rehabilitating their fingers and thus improve the quality of life of patients. Researchers have studied elastomer‐based soft robotic gloves driven by pneumatic powers.^86^, ^87^ The compliance of the soft materials allows the gloves to closely fit the hands. Some development works have been conducted to further enhance the functionalities of the elastomer‐based soft robotic gloves, such as using EMG signals to assist rehabilitation,^89^ or adopting variable stiffness designs for better compliance.^86^ In addition to elastomer materials, researchers have also developed soft robotic gloves based on other materials such as fabrics^75^, ^76^ and soft 3D printing materials.^87^ Besides pneumatic‐driven soft robotic gloves, some other actuation mechanisms are also adopted for soft robotic gloves, such as SMA^90^ and cable‐driven mechanism.^87^

Soft robotic exosuits are primarily used for back or shoulder rehabilitations. D. Govin et al. reported a research work on soft robotic back orthosis.^91^ This back orthotic device can assist patients to achieve a fully upright position of the back and stabilize the lumbosacral spine by using pneumatic‐driven soft bladders made from fabrics. Another soft robotic exosuit for waist support during lifting tasks is an active soft waist exoskeleton driven by pneumatic muscles to support users during lumbar‐based activities such as stoop lifting.^92^ Natividad et al. reported soft exosuits toward shoulder rehabilitation.^93^ Modular soft pouches made from thermoplastic polyurethane (TPU) fabrics were used to fabricate the exosuits. The suit is designed to support arm motion in two degrees of freedom, abduction, and horizontal flexion, with a low pneumatic pressure requirement (∼10 kPa).

Soft robotic exosuits can also provide gait rehabilitation and mobility aids. Awad et al. investigated the effects of a portable soft robotic exosuit on individuals post‐stroke rehabilitation.^94^ The exosuit provides plantar flexor force assistance during the stance phase and helps dorsiflexors during the swing phase to reduce drop‐foot. The results suggest that lightweight and nonrestrictive exosuit soft robotic exosuits can help post‐stroke individuals achieve meaningful increases in speed and distance. In another work,^79^ a soft robotic exosuit was designed to supplement the partially paralyzed lower limb's ability to generate forward movement. The exosuit, when powered on, significantly boosts ankle dorsiflexion, reduces interlimb propulsion asymmetry, and reduces the energy required to walk during treadmill and overground walking tests. Sridar et al.^78^, ^95^ propose a soft‐inflatable exosuit using heat‐sealable TPU materials to aid the knee extension during the stroke patient rehabilitation training process. Inertial measurement units and smart shoe insole sensors embedded force‐sensitive resistors are integrated to improve gait phase detection and controller design, and soft‐inflatable actuators are characterized to generate the necessary stiffness outputs. Reduced muscle activity is observed, which proves that this lightweight, low‐budget, and body‐conforming interface soft‐inflatable exosuit can potentially be used for individuals with impairment.

## 6 CHALLENGES AND FUTURE DIRECTIONS

Soft robotic devices for biomedical applications face several research challenges in the aspects of long‐term stability, energy sources, and roadmap translating from lab to clinical usage. As discussed in the previous sections, soft robotic devices have been extensively explored and studied for surgical intervention, rehabilitation and assistance, bio‐signal sensing, and soft implantation. However, most of the state‐of‐art works are still in exploration or clinical trial stages and might be years away from wide adoption in real applications. To move forward, easy integration with biological systems or the human body is crucial. For example, wearables, implants, and drug delivery devices need to be portable or untethered. Thus, reliable power sources with long‐lasting, lightweight, quiet, and safe features would be significant in the widespread adoption of soft robotic devices. For example, fluid‐driven soft actuators need sources of compressed air or hydraulic flow, while air compressors and hydraulic pumps are usually rigid, heavy, bulky, and noisy. This has become a major technological barrier for fluidic driven soft robotic devices to be applied in biomedical applications. In addition, some soft actuation technologies require or generate high temperature, strong electric fields or high currents, which results in frequent replacement of batteries and safety concerns. Future approaches to address these issues could be employing the human energy sources (such as adenosine triphosphate) to drive wearable devices or implants. Multi‐disciplinary research involving biology, materials science, chemistry, and robotics will be necessary to achieve such power sources. In addition, future development of portable soft pumps and batteries made of soft materials will also pave the way for safer and more comfortable soft robotic devices for healthcare use.

Long‐term stability and durability are also major challenges that need to be addressed for the future development of biomedical soft robotic devices, as both internal and external environment of the human body defines high requirements of long‐term stability and durability. For example, the acidic environment of the stomach may cause material aging and structural failure of soft endoscopes and manipulators. Wearable soft robotic devices are usually subjected to repeating cyclic movements and may cause cracks to develop on the soft materials. In addition, resistance to ultraviolet light or high temperature is also required due to sanitization. As a result, huge research efforts are needed to develop soft materials with high robustness, high durability, and long‐term stability, making them able to adapt to both internal and external human body environments. In addition, self‐healing soft materials allow the soft robot to repair its damaged parts for a longer time of usability, and advancing manufacturing techniques will also improve the stability of soft robotic devices. Current fabrication methods include mold casting, coating and 3D printing, allowing the realization of disparate soft structures. However, weak interfaces created between the print layers and soft‐to‐rigid interfaces when fabricating complex geometries become the limiting factor to robust and durable soft robotic devices.

In the near future, multidisciplinary research is needed for innovative solutions in the field of engineering, mechanics, materials science, biological science, and even artificial intelligence.^96^ There is still a long way for soft robotic devices to be clinically effective, safe, and stable to finally enable real clinical translation. As a future perspective, soft robotics has paved the way for innovative solutions that have a significant impact on changing the landscape of healthcare and translational medicine in the near future. This is an extremely emerging and attractive field, and the realization of soft robots working closely with patients and clinicians needs innovation and enthusiasm to find solutions to address the research challenges toward the clinical approval of healthcare devices.

## AUTHOR CONTRIBUTIONS

Yuzhe Wang conceived the idea; Yuzhe Wang, Zhen Xie, Huishi Huang and Xinquan Liang wrote the original manuscript; Yuzhe Wang revised the manuscript.

## CONFLICT OF INTEREST STATEMENT

All authors declare no conflicts of interest.
